# Investigation on factors related to poor CPAP adherence using machine learning: a pilot study

**DOI:** 10.1038/s41598-022-21932-8

**Published:** 2022-11-15

**Authors:** Kana Eguchi, Tsutomu Yabuuchi, Masayuki Nambu, Hirofumi Takeyama, Shozo Azuma, Kazuo Chin, Tomohiro Kuroda

**Affiliations:** 1 NTT Smart Data Science Center, Nippon Telegraph and Telephone Corporation, Tokyo, Japan; 2 NTT Human Informatics Laboratories, Nippon Telegraph and Telephone Corporation, Kanagawa, Japan; 3 Preemptive Medicine and Lifestyle-Related Disease Research Center, Kyoto University Hospital, Kyoto, Japan; 4 Department of Respiratory Care and Sleep Control Medicine, Graduate School of Medicine, Kyoto University, Kyoto, Japan; 5 Division of Medical Information Technology and Administration Planning, Kyoto University Hospital, Kyoto, Japan; 6 Present Address: Department of Real World Data R&D, Graduate School of Medicine, Kyoto University, Kyoto, Japan; 7 Present Address: Department of Neurology, Japanese Red Cross Otsu Hospital, Shiga, Japan; 8 Present Address: Center for Genomic Medicine, Graduate School of Medicine, Kyoto University, Kyoto, Japan; 9 Present Address: Division of Sleep Medicine, Department of Sleep Medicine and Respiratory Care, Department of Internal Medicine, Nihon University of Medicine, Tokyo, Japan

**Keywords:** Prognosis, Respiratory tract diseases, Therapeutics, Information technology

## Abstract

To improve patients’ adherence to continuous positive airway pressure (CPAP) therapy, this study aimed to clarify whether machine learning-based data analysis can identify the factors related to poor CPAP adherence (i.e., CPAP usage that does not reach four hours per day for five days a week). We developed a CPAP adherence prediction model using logistic regression and learn-to-rank machine learning with a pairwise approach. We then investigated adherence prediction performance targeting a 12-week period and the top ten factors correlating to poor CPAP adherence. The CPAP logs of 219 patients with obstructive sleep apnea (OSA) obtained from clinical treatment at Kyoto University Hospital were used. The highest adherence prediction accuracy obtained was an F1 score of 0.864. Out of the top ten factors obtained with the highest prediction accuracy, four were consistent with already-known clinical knowledge. The factors for better CPAP adherence indicate that air leakage should be avoided, mask pressure should be kept constant, and CPAP usage duration should be longer and kept constant. The results indicate that machine learning is an adequate method for investigating factors related to poor CPAP adherence.

## Introduction

Obstructive sleep apnea syndrome (OSAS) is a highly prevalent sleep disorder that can cause sleep deprivation and excessive daytime sleepiness. OSAS is characterized by repetitive episodes of partial or complete upper airway obstruction during sleep^1^. Treatment is crucial because OSAS is known to negatively impact quality of life (QoL)^1^ and is associated with an increased risk of cardiovascular disease^2,3^. Continuous positive airway pressure (CPAP) therapy^4^ is the standard treatment for OSAS^5^. Although CPAP therapy is known to suppress excessive daytime sleepiness, improve QoL^6^, and decrease the risk of cardiovascular outcomes^7,8^, a significant proportion of patients underuse or discontinue CPAP^9^. Therefore, ensuring CPAP adherence still remains an important clinical issue. Because CPAP machines can record several usage-related parameters on a daily basis (see “Preprocessing” section for details), Schwab et al.^10^ stated that studies should examine the usefulness of the CPAP logs and how the parameters affect OSA outcomes.

However, the relationship between CPAP usage-related parameters and CPAP adherence has yet to be investigated. Most clinical studies have only evaluated a single factor or a few factors to determine a method for ensuring CPAP adherence^5,10–14^. Meanwhile, machine learning-based data analysis has been gaining attention; using a large amount of data, machine learning enables predictive analytics while uncovering hidden patterns and unknown correlations. Several studies have shown its usefulness for the management of chronic disease treatment. For example, a study targeting the hospital visits of diabetic patients^15^ revealed factors possibly correlated to missed scheduled clinical appointments while also predicting whether a target patient will miss a scheduled clinical appointment. Although Araujo et al.^16^ and Scioscia et al.^17^ applied machine learning to CPAP logs, their studies focused on predicting CPAP adherence rather than investigating usage-related factors associated with adherence.

In our study, we aim to clarify whether machine learning-based data analysis is effective for identifying CPAP usage-related parameters associated with adherence while also predicting adherence. In particular, we focus on revealing factors possibly correlated to poor adherence which can be avoided.

## Methods

We designed a machine learning model to verify the aforementioned hypothesis. Our study uses the same model for different purposes in its training phase and test phase. In the training phase, we use the model with a huge amount of training data (e.g., previously collected CPAP logs from many individuals) to investigate factors possibly correlated to poor CPAP adherence. In the test phase, we use the model with recent CPAP logs of a target patient to predict whether this patient’s CPAP adherence will become poor within a target period.

On the basis of the definition by the Centers for Medicare and Medicaid Services (CMS)^5^, this study defines good adherence as usage of the CPAP machine for more than four hours per night and more than five nights a week (i.e., 70% of nights in a week), whereas poor adherence is not good CPAP adherence.

The proposed model comprises two parts: preprocessing and a machine learning-based model. The details of each part are described below.

### Preprocessing

Preprocessing comprises feature calculation and data standardization.

First, a total of eighteen weekly features are calculated as shown in Table 1. Although a CPAP machine provides a set of measured data per day, in this study we calculate all features from every seven consecutive days to suppress night-to-night variability. The CPAP machine used in this study was made by ResMed (ResMed Inc., San Diego, CA, USA). The daily recorded data comprises two qualitative values (sex and CPAP mode: auto-titration (auto) or fixed-titration) and five quantitative values (usage duration, air leakage, apnea index (AI), apnea hypopnea index (AHI), and daily average mask pressure). In addition to the original qualitative values provided by the CPAP machine, we calculate two additional qualitative features, daily severity based on AHI (Normal, Mild, Moderate, Severe)^18^ and the daily presence of OSAS based on AI as defined by Guilleminault et al.^19^. To calculate these two qualitative features, we first identify the daily value of each one and then set the most frequent value in a week as the weekly feature value.

**Table 1:** List of features obtained from CPAP logs.

Number	Feature name	Unit	Value
	**Qualitative values obtained from the original CPAP log**		
1	Sex		Male/female
2	CPAP mode (i.e., pressure control)		Auto/fixed
	**Summaries of quantitative values obtained from CPAP log: usage duration**		
3	Percentage of days in a week with more than 4 h of CPAP usage	%	
4	Percentage of days in a week with CPAP usage	%	
5	Percentage of days in a week without CPAP usage	%	
6	Average daily usage duration in a week	min	
7	Standard deviation of daily usage duration in a week	min	
8	Total usage duration in a week	min	
	**Summaries of quantitative values obtained from CPAP log: air leakage**		
9	Average daily air leakage from CPAP mask in a week	L/sec	
10	Standard deviation of daily air leakage from CPAP mask in a week	L/sec	
	**Summaries of quantitative values obtained from CPAP log: AI**		
11	Average daily AI in a week	events/h	
12	Standard deviation of daily AI in a week	events/h	
	**Summaries of quantitative values obtained from CPAP log: AHI**		
13	Average daily AHI in a week	events/h	
14	Standard deviation of daily AHI in a week	events/h	
	**Summaries of quantitative values obtained from CPAP log: average mask pressure (from auto mode only)**		
15	Average of daily average mask pressure in a week	cmH_2_O	
16	Standard deviation of daily average mask pressure in a week	cmH_2_O	
	**Additional qualitative values**		
17	Severity of OSAS^18^		Normal/Mild/Moderate/Severe
18	Presence of OSAS^19^		Normal (AI < 5)/OSAS (5 ≤ AI)

*CPAP* continuous positive airway pressure; *OSAS* obstructive sleep apnea syndrome; *AI* apnea index; *AHI* apnea–hypopnea index.

The last step of preprocessing is data standardization. When targeting several different types of quantitative values, the difference in each data range causes different updating volumes of the weight corresponding to each feature in the training phase. Data standardization converts the data to a standardized value with the same scale. We use Eq. (1) to calculate the standardized value *ã*, whose mean value is 0 and standard deviation is 1. In Eq. (1), *μ* denotes the mean value of feature *a*, whereas *σ* denotes its standard deviation.

1 $$\tilde{a} = \frac{a - \mu }{\sigma }$$

### Machine learning-based model

We built a prediction model using logistic regression and a learn-to-rank (LTR) machine learning algorithm with a pairwise approach^20^. In the training phase, the proposed model aims to rank all patients in accordance with the risk of poor CPAP adherence, giving a higher rank to a patient whose CPAP adherence worsens within a shorter period. Through this ranking process, the proposed model calculates a weight vector that indicates the correlation between each parameter and poor CPAP adherence. In the test phase, the proposed model with the calculated weight vector identifies the target patient among all patients used in the training phase. Because the order of patients reflects the number of weeks until poor CPAP adherence, we can predict whether the target patient’s CPAP adherence becomes poor within a target period by determining a threshold for the rank. The details of the model are described below step by step.

#### Auxiliary parameter used for model training

Ensuring a sufficient amount of data for the training is quite important when applying machine learning for a limited amount of data. In general, we cannot use data shorter than the target period. Because of this, we may easily result in unsuccessful predictions due to a lack of training data.

Making the best of the limited number of CPAP usage logs for training the model, this study uses the number of weeks of each patient’s CPAP adherence as an auxiliary parameter. This duration is calculated by either of the following two. The first is the number of weeks until the first instance of poor CPAP adherence *PA (p*_*x*_*, t*_*x*_*)*, where *p*_*x*_ is the patient and *t*_*x*_ is the number of weeks from the first week until the week when poor CPAP adherence was observed for the first time. When a patient *p*_*x*_ exhibits poor CPAP adherence, we calculate *PA (p*_*x*_*, t*_*x*_*)*. The second is the number of weeks of continuous good CPAP adherence *GA (p*_*y*_*, t*_*y*_*)*, where *p*_*y*_ is the patient and *t*_*y*_ is the number of weeks from the first week until the most recent week when good CPAP adherence was observed. When a patient *p*_*y*_ was able to continuously maintain good adherence without exhibiting poor CPAP adherence, we calculated *GA (p*_*y*_*, t*_*y*_*)*.

#### Basic design of logistic regression model

This study modeled the probability *y*_*m,n*_ that the CPAP adherence of patient *p*_*m*_ at time *t*_*m*_ would become poor earlier than another patient *p*_*n*_ at *t*_*n*_ using logistic function expressed as Eq. (2).

2 $$P\left( {y_{m,n} {|}x_{m} ,x_{n} ;w} \right) = \frac{1}{{1 + \exp \left( { - yw \cdot \left( {x_{m} - x_{n} } \right)} \right)}}$$

here, $w$ is a weight vector, and *x*_*m*_ and *x*_*n*_ are feature vectors of patients *p*_*m*_ and *p*_*n*_, respectively.

Mathematically, the logit of Eq. (2) is expressed as Eq. (3).

3 $$logit\left( {P\left( {y_{m,n} {|}x_{m} ,x_{n} ;w} \right)} \right) = \log \left( {\frac{{P\left( {y_{m,n} {|}x_{m} ,x_{n} ;w} \right)}}{{1 - P\left( {y_{m,n} {|}x_{m} ,x_{n} ;w} \right)}}} \right) = yw \cdot \left( {x_{m} - x_{n} } \right)$$

Because the logit is the inverse function of the logistic function, the logit value indicates the probability: logit(*P*) < 0 indicates *P* < 0.5, logit(*P*) = 0 indicates *P* = 0.5, and logit(*P*) > 0 indicates *P* > 0.5. Therefore, we substitute *y* in Eqs. (2) and (3) for either +1 or − 1 depending on the duration until poor CPAP adherence, i.e., *y* = +1 when *PA (p*_*m*_*, t*_*m*_*)* is shorter than *PA (p*_*n*_*, t*_*n*_*)* or when *PA (p*_*m*_*, t*_*m*_*)* is shorter than *GA (p*_*n*_*, t*_*n*_*)*, and *y* = − 1 when *PA (p*_*m*_*, t*_*m*_*)* is longer than *PA (p*_*n*_*, t*_*n*_*)*.

#### Model design and training with L2-norm regularization

The aim of the training process is to rank all patients in the training data in accordance with the duration until poor CPAP adherence by repeating the ranking on every pair of patients.

For this ranking, the model uses the logit value *w*∙*x*_*m*_ as the PA risk score to measure the risk of a patient *p*_*m*_’s CPAP adherence becoming poor at *t*_*m*_. Here, the PA risk score is a specific case of Eq.﻿ (3) assuming *y* = +1 while setting an imaginary patient whose feature *x*_*n*_ is 0. In other words, the proposed model aims to rank all patients in the training data through comparison with the same imaginary patient. This ranking is only conducted in the following two cases: when *PA (p*_*m*_*, t*_*m*_*)* is shorter than *PA (p*_*n*_*, t*_*n*_*)*, and when *PA (p*_*m*_*, t*_*m*_*)* is shorter than *GA (p*_*n*_*, t*_*n*_*)*. Through the training process, the model calculates *w* to match the value of the PA risk score and the number of weeks until poor CPAP adherence, giving a larger PA risk score to the earlier occurrence of poor CPAP adherence.

To mitigate overfitting to the training data set and improve the model’s generalizability for the new data, we used an L2-norm regularization as in the previous study^15^. The model estimates *w* as $\hat{w}$ by using the following equation.

4 $$\hat{w} = \arg \max \left\{ {\mathop \sum \limits_{n = 1}^{N} \log P\left( {y_{m,n} {|}x_{m} ,x_{n} ;w} \right) - \lambda w_{2}^{2} } \right\}$$

here, $w_{2}^{2}$ is an L2-norm regularizer (i.e., the squared L2-norm of *w*), which acts as a penalty to provide large absolute weight values for certain features that occur frequently in the training data. The symbol λ is a hyperparameter for regularization. We need to tune hyperparameter λ while comparing the performance of the model.

#### Predicting CPAP adherence using proposed model

In the test phase, the model estimates CPAP adherence based on the PA risk score of the target patient using the estimated $\hat{w}.$ Because we designed the PA risk score to reflect the number of weeks until poor CPAP adherence, we can predict whether the target patient’s CPAP adherence becomes poor within a target period by determining a threshold for the PA risk score.

## Evaluation

We applied our model to the CPAP logs of the participants to predict whether their CPAP adherence became poor within twelve weeks (approximately three months). On the basis of a weight vector calculated through the training phase, we investigated the CPAP usage-related parameters possibly correlated with poor adherence.

In this study, we obtained the patient data with the opt-out consent, which is specified by Article 8-1-(2)-"a"-"i" of Ethical Guidelines for Medical and Biological Research Involving Human Subjects of Japan. We posted the introduction of this evaluation on a webpage and set a waiver period. During this period, all participants were eligible to opt-out of this evaluation by declaring their waiver. All evaluations were conducted in accordance with the protocol approved by the Ethics Committee of Kyoto University Hospital (R1821).

### Participants

A total of 354 patients who used CPAP machines (ResMed Inc, San Diego, CA, USA) from January 1, 2007 to December 31, 2018, were eligible for this retrospective study. Note that their OSAS diagnosis criterion was an AHI greater than 20 in polysomnography reports.

**Figure 1:**
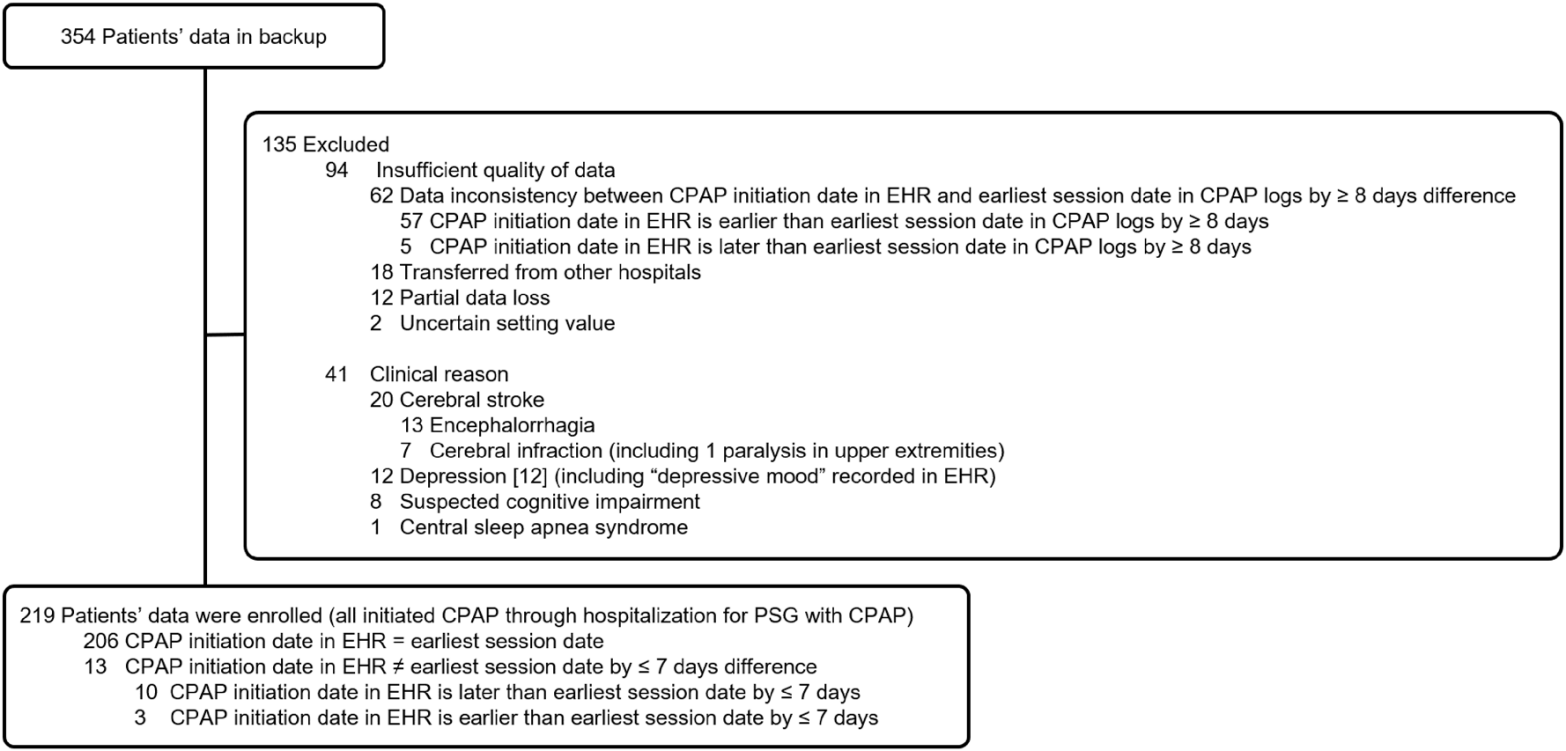
Flowchart of participant selection. Definition of abbreviations: CPAP = continuous positive airway pressure; EHR = electric health record.

The exclusion criteria included insufficient data quality and certain diseases (e.g., depression^12^), as shown in Fig. 1. Note that this study excluded participants transferred from other hospitals as a part of insufficient data quality because we cannot calculate an auxiliary parameter due to the lack of a CPAP initiation date.

After excluding participants on the basis of the aforementioned criteria, 219 patients were selected. Table 2 summarizes the clinical background of these participants.

**Table 2:** Clinical background of 219 participants and their CPAP usage characteristics during CPAP therapy.

Variable	Unit	Value
**Clinical background**		
Number of patients		219
Male	%	86.8 (N = 190)
Age at CPAP start	Year	61.5 ± 12.0
Body mass index	kg/m^2^	27.5 ± 5.46
AHI at PSG at diagnosis	events/h	47.3 ± 18.1
AHI at PSG at CPAP start	events/h	7.34 ± 8.45
**CPAP usage characteristics in CPAP logs**		
CPAP mode (auto/fixed)	%	98.2/1.80
Daily mean mask pressure in auto mode	cmH_2_O	8.57 ± 2.02
Daily usage duration	min	334 ± 121
Daily AI	events/h	1.35 ± 2.43
Daily AHI	events/h	2.03 ± 2.74
Daily air leakage	L/sec	0.10 ± 0.15

Data represent mean ± standard deviation. *CPAP* continuous positive airway pressure; *AHI* apnea–hypopnea index; *PSG* polysomnography; *AI* apnea index.

### Data used for evaluation

The CPAP logs obtained through the clinical CPAP treatment were used for retrospective data analysis. We only used the data from nights when CPAP was used for more than thirty minutes. This is because AI and AHI can be erroneous when the CPAP usage duration is short as AI and AHI are indices showing the target event per hour.

**Figure 2:**
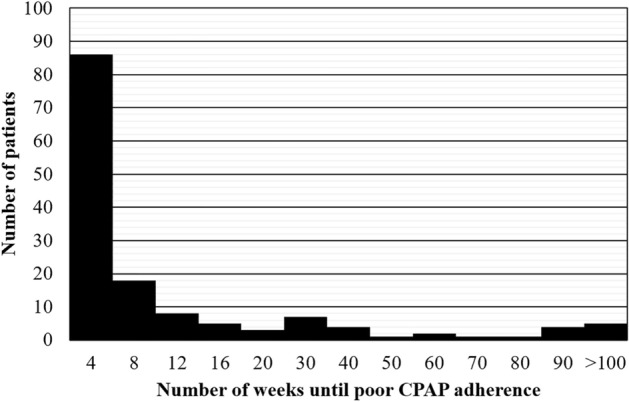
Number of weeks until first poor CPAP adherence among 145 patients who exhibited poor CPAP adherence.

Regarding the CPAP adherence of the target 219 patients, we confirmed that two patients had *GA* (i.e., 8 and 101 weeks), 145 patients had *PA*, and the remaining 72 patients experienced poor CPAP adherence in the first week and cannot calculate either *PA* or *GA*. The histogram of the number of weeks until the first poor CPAP adherence among 145 patients who had *PA* is shown in Fig. 2. Technically, the following evaluation only targets the data of 147 patients who had *GA* or *PA*.

### Evaluation methods

We evaluated the results in terms of the prediction performance of CPAP adherence and the top ten CPAP usage-related parameters possibly correlated with poor CPAP adherence.

Before investigating both the prediction performance and the top ten CPAP usage-related parameters possibly correlated with poor CPAP adherence, technically, we need to determine the hyperparameter λ that optimizes ranking performance. As a pre-evaluation, this study evaluated whether the model correctly ranked the participants in the test dataset using five-fold cross-validation. The k-fold cross-validation is a statistical validation method, where *k* is a user-specified number (usually 5 or 10)^21^. When performing five-fold cross-validation, the data is first partitioned into five subsets of approximately equal size, and then a sequence of models is trained and tested five times. For each test, one of the subsets is used as the test data and the rest of the four subsets are used for the training data. After all trials, the accuracy is obtained as the average across all five trials. To determine the appropriate hyperparameter λ, we evaluated seven different hyperparameters (λ = 0.1, 0.2, 0.5, 1.0, 2.0, 5.0, 10.0) in each cross-validation test.

After determining the hyperparameter λ, we evaluated the prediction performance of CPAP adherence using the PA risk scores and their corresponding duration until poor CPAP adherence. For this evaluation, we used the receiver operating characteristic (ROC) curve^22^ plotted in 0.5 increments of the PA risk score.

Regarding the top ten CPAP usage-related parameters possibly correlated with poor CPAP adherence, this study investigated a weight vector $\hat{w}$ calculated with the optimal hyperparameter λ. Because a larger absolute value indicates a stronger correlation, we selected the features with the ten largest absolute value weights as the top ten factors and evaluated whether they are consistent with common clinical knowledge.

## Results

### Pre-evaluation on ranking accuracy for determining Hyperparameter λ

The ranking accuracy of our model was 0.692 ± 0.010 and reached the highest at 0.706 when the hyperparameter λ = 0.2. Therefore, we set the hyperparameter λ = 0.2 for the following evaluation.

### Prediction performance

**Figure 3:**
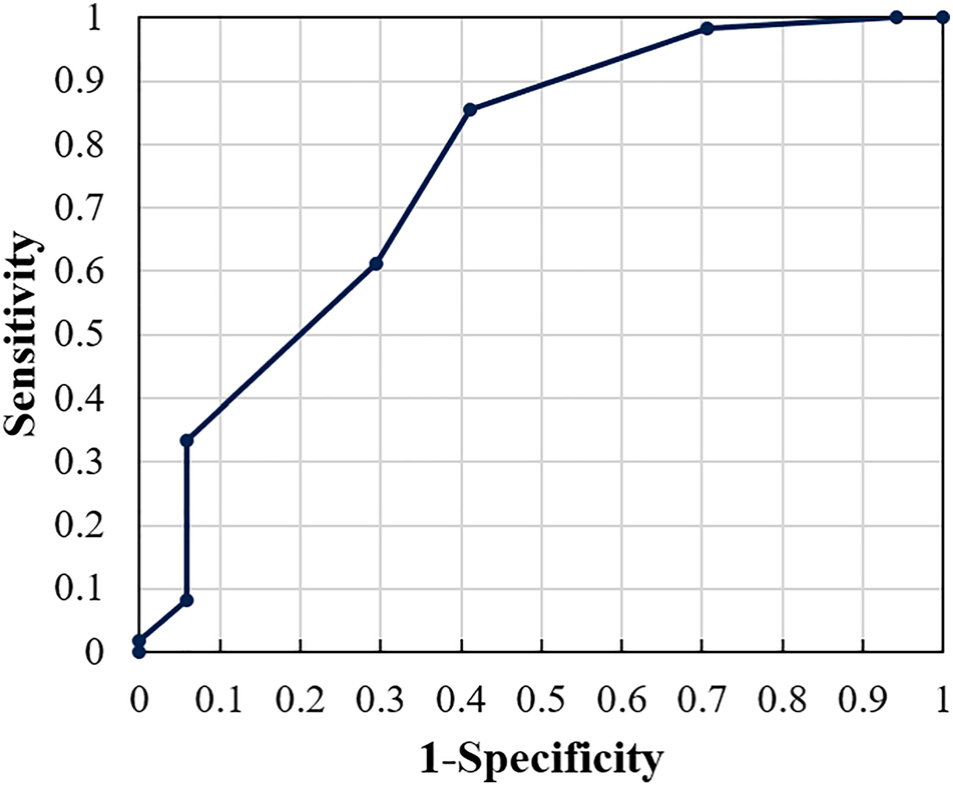
Receiver operating characteristic (ROC) curve indicating prediction performance of proposed model.

Figure 3 shows the ROC curve. The area under the curve (AUC) of the ROC was estimated as 0.763, indicating moderate accuracy^22^. The optimal cut-off point of the PA risk score based on the Youden index^22^ was − 0.5, where the F1 score was 0.864, precision was 0.872, and recall was 0.856, respectively.

### CPAP usage-related factors related to poor adherence

Table 3 lists the top ten CPAP usage-related parameters possibly correlated with poor CPAP adherence and their interpretations obtained from the highest ranking accuracy (hyperparameter λ = 0.2). For the weights shown in Table 3, a positive number indicates a positive correlation and a negative number indicates a negative correlation. For example, the top factor, average duration of usage in a week, has a strong negative correlation (− 9.38) with poor CPAP adherence. This indicates that participants using CPAP for shorter durations may have poor CPAP adherence in the future.

**Table 3:** List of top ten factors possibly correlated to poor CPAP adherence obtained with highest ranking accuracy.

No	Feature name	Weight	Implications	Interpretation
1	Average duration of daily usage in a week	− 9.38	Participants who used CPAP for shorter hours may have poor CPAP adherence	Feasible. Consistent with^13^
2	Total duration of usage in a week	− 8.89	Participants using CPAP for shorter hours may have poor CPAP adherence	Feasible. Consistent with^13^
3	Auto mode	5.14	Participants using CPAP in auto mode may have poor CPAP adherence	Possibly biased (auto = 98.2%)
4	Female	4.82	Female participants may have poor CPAP adherence	Possibly biased (female = 13.2%)
5	Standard deviation of daily average mask pressure in a week (from auto mode only)	4.75	Participants whose CPAP mask pressure varies greatly in a week may have poor adherence	Feasible
6	Standard deviation of daily usage duration in a week	4.66	Participants whose CPAP usage duration varies greatly in a week may have poor CPAP adherence	Feasible
7	Average daily AHI in a week	4.16	Participants who had higher AHI may have poor CPAP adherence	Feasible. Consistent with^14^
8	Presence of OSAS was “normal”	3.72	Participants whose AI was less than five (“normal”) may have poor CPAP adherence	Possibly biased (“normal” = 96.3%)
9	Average daily air leakage in a week	3.26	Participants who experienced larger air leakage from a CPAP mask may have poor CPAP adherence	Feasible. Consistent with^14^
10	Severity of OSAS was “normal”	3.13	Participants whose AHI was less than five (“normal”) may have poor CPAP adherence	Possibly biased (“normal” = 92.8%)

*CPAP* continuous positive airway pressure; *AHI* apnea–hypopnea index; *AI* apnea index; *OSAS* obstructive sleep apnea syndrome.

## Discussion

To our knowledge, our study is the first to use machine learning to investigate CPAP usage-related parameters possibly correlated with poor CPAP adherence while also predicting future CPAP adherence. We developed a model using logistic regression and LTR machine-learning algorithm with a pairwise approach^20^, then applied it to the CPAP logs obtained from clinical treatment in one hospital. Overall, the results indicated that our model can be a fair method for both predicting CPAP adherence and investigating the factors correlated to poor adherence.

Regarding adherence prediction performance, our model using one week of CPAP logs was able to predict the risk of poor CPAP adherence within twelve weeks with an AUC value of 0.763, and its prediction performance at the optimal cut-off point yielded an F1 score of 0.864. Compared with the results of a previous study^16^, which used 13 days (approximately two weeks) of CPAP logs for predicting the risk of poor adherence within 180 days (approximately 26 weeks after) with an F1 score of 0.54, our proposed model showed good performance.

We identified six factors regarding CPAP usage-related parameters possibly correlated to poor adherence. Among these six factors, four were consistent with previous clinical knowledge^13,14^: Factors 1 and 2 are consistent with the discussion presented by Chai-Coetzer et al.^13^, and Factors 7 and 9 are consistent with the discussion by Valentin et al.^14^. Thus, the results of our pilot study indicate that machine learning is an adequate method for investigating factors related to poor CPAP adherence. Overall, the feasible factors identified by this retrospective study indicated that the following would improve CPAP adherence: avoiding air leakage, keeping a constant mask pressure, and longer and constant CPAP usage duration. Meanwhile, a detailed analysis of the data used in this study indicated that the remaining four factors may be strongly biased. For Factor 3, 98.2% of the data were obtained from auto mode; for Factor 4, 86.8% of the data were from men; for Factor 8, 96.3% of data were classified as “normal”; and for Factor 10, 92.8% of data were classified as “normal.” In theory, machine learning may overvalue a feature when it repeatedly shows the same tendency; for example, machine learning may overvalue women more than men if the CPAP adherence in female patients is consistently poor, whereas it varies among male patients. At this moment, we cannot determine whether these four factors are surely correlated with poor CPAP adherence. Further studies on additional patients will be needed to clarify the relationship between the four factors and CPAP adherence. It should be noted that these imbalanced conditions were quite common under clinical OSAS treatment settings; these biases simply indicate that the applied dataset was not eccentric but can be considered a valid subset of Japanese real-world data.

To improve prediction accuracy, we need to modify the model in consideration of real-world CPAP usage. In this study, our model focuses on predicting poor CPAP adherence using weekly features. Although weekly features could reflect basic characteristics of night-to-night CPAP usage, they may unintentionally suppress specific characteristics that occurred within a week (e.g., temporarily discontinuation of CPAP use subsequent to the specific CPAP use in the previous night). In addition, the current model does not consider how to handle data that resumes after discontinuation (e.g., trip) as well as partial data (e.g., hospital transfer). In addition, the model may also need to consider the date of CPAP use. Physicians have pointed out that CPAP adherence can change depending on the season (e.g., adherence may worsen in the change of season from winter to spring due to pollinosis). Future studies should consider these perspectives to clarify CPAP use and improve adherence.

The main limitation of this research is the lack of causal relation analysis, which is a common limitation in retrospective research. Since this study was a pilot and retrospective study, the results only indicate correlations. Therefore, we first intend to verify whether the factors indicated in this study can improve clinical CPAP adherence through a prospective study. Once the relationship between the factors and clinical CPAP adherence has been confirmed, patients of CPAP therapy can be advised on the basis of these parameters. Another limitation of our study is the small scale. All aforementioned implications were obtained from retrospective data analysis using previously collected clinical CPAP logs from only one hospital, so the calculated weight vector only conceals characteristics inherent in this data theoretically. To verify the consistency of the results, a large-scale study including other hospitals is necessary for deeper investigation.

## Conclusion

We developed a CPAP adherence prediction model using logistic regression and learn-to-rank machine learning, and then applied it to CPAP logs obtained from clinical treatment provided by one hospital. The results of retrospective data analysis indicate that machine learning is sufficient for investigating factors related to poor CPAP adherence. The factors obtained from retrospective data analysis support previous clinical knowledge for improving CPAP adherence.

In general, machine learning can be considered a promising method for uncovering medical knowledge. Although this study did not provide new findings, it shows the potential of machine learning for achieve the prospective goal. Towards this end, we will need to collect a larger amount of data with various parameters and apply machine learning predictors for deeper investigation.

## Data Availability

The datasets generated and/or analyzed during the current study are not publicly available due to Articles 27 and 28 of the Japanese Personal Information Protection Law that prohibits any "Personal Information Handling Business Operators" to share any personal data including "Special Care-Required Personal Information" like medical history without obtaining in advance a principal's consent, but are available from the corresponding author on reasonable request following Article 20-2-5 of the Personal Information Protection Law.

## Code availability

Not applicable (cannot share the source file of program code due to the protection of business secrecy).
